# Genome-wide analyses of the prognosis-related mRNA alternative splicing landscape and novel splicing factors based on large-scale low grade glioma cohort

**DOI:** 10.18632/aging.103491

**Published:** 2020-07-13

**Authors:** Wang-Rui Liu, Chuan-Yu Li, Wen-Hao Xu, Xiao-Juan Liu, Hai-Dan Tang, Hai-Neng Huang

**Affiliations:** 1 Department of Neurosurgery, Affiliated Hospital of Youjiang Medical University for Nationalities, Guangxi 533000, China; 2 Clinical College of Youjiang Medical University for Nationalities, Baise 533000, China; 3 Department of Urology, Fudan University Shanghai Cancer Center, Shanghai 200032, China; 4 Department of Oncology, Shanghai Medical College, Fudan University, Shanghai 20032, China; 5 Department of Pathogenic Biology, Medical College, Nantong University, Nantong 226001, Jiangsu, China; 6 Department of Neurology, Affiliated Hospital of Youjiang Medical College for Nationalities, Guangxi 533000, China

**Keywords:** alternative splicing, bioinformatics, low grade glioma, prognosis, splicing factors

## Abstract

Alternative splicing (AS) changes are considered to be critical in predicting treatment response. Our study aimed to investigate differential splicing patterns and to elucidate the role of splicing factor (SF) as prognostic markers of low-grade glioma (LGG). We downloaded RNA-seq data from a cohort of 516 LGG tumors in The Cancer Genome Atlas and analyzed independent prognostic factors using LASSO regression and Cox proportional regression to build a network based on the correlation between SF-related survival AS events. We collected 100 patients from our center for immunohistochemistry and analyzed survival using χ2 test and Cox and Kaplan-Meier analyses. A total of 9,616 AS events related to LGG were screened and identified as well as established related models. Through analyzing specific splicing patterns in LGG, we screened 16 genes to construct a prognostic model to stratify the risk of LGG patients. Validation revealed that the expression level of the prognostic model in LGG tissue was increased, and patients with high expression showed worse prognosis. In summary, we demonstrated the role of SFs and AS events in the progression of LGG, which may provide insights into the clinical significance and aid the future exploration of LGG-associated AS.

## INTRODUCTION

Glioma is a tumor of the central nervous system caused by abnormal growth of glial cells [1]. Pathological anatomy shows that it is mainly composed of astrocytes and oligodendrocytes. The World Health Organization (WHO) classifies gliomas into four grades [2]: Grade I and II are low-grade gliomas (LGGs) and Grade III/IV are high-grade gliomas (HGGs). LGG usually grows slowly, and accounts for approximately 20% of all primary brain tumors [2]. Studies have shown that LGG survival rate is higher than that of HGG; the median survival time of LGG patients is between 5 and 10 years, and the survival time of some subtypes can exceed 10 years [3]. Although LGG patients have a longer survival time than HGG patients, it is concerning that many patients will deteriorate from LGG to HGG approximately 4–5 years after diagnosis [4, 5], resulting in a sharp decline in prognosis. Although the surgical methods and pre- and post-operative treatments for LGG are often controversial in the field of neurosurgery, doctors are unified in the purpose of treatment, that is, to extend overall survival (OS) while maintaining the quality of life of patients [6]. Therefore, how to delay the progress of LGG and prevent its deterioration to HGG has become the latest research direction. Unfortunately, there are still few biomarkers available that accurately reflect the progression of LGG, and thus new biomarkers are urgently required to monitor LGG.

Alternative splicing (AS) is a mechanism that allows cells to generate a considerable amount of protein diversity from a limited number of genes [7]. It is important to regulate the transcription of mRNA through transcription regulation mechanisms [8]. AS events can be divided into seven types, and each has a different mechanism of action: alternate acceptor site (AA), alternate donor site (AD), alternate promotor (AP), alternate terminator (AT), exon skip (ES), mutually exclusive exons (ME), and retained intron (RI). Recent research showed that abnormal AS events have important roles in cancer including in tumor progression, metastasis, endogenous processes, and resistance to treatment [9–12]. Mutations in splicing factor (SF) expression may not only cause the activation of oncogenes or cancer pathways but may also be related to the loss of tumor suppressor function [13, 14]. Since AS events are inextricably linked to cancer, their executors, SF genes, may be potential targets for cancer treatment programs.

In the current study, we obtained RNA-seq from The Cancer Genome Atlas (TCGA) program and elucidated the role of differential AS patterns in 516 LGG cohorts to conduct an in-depth analysis of the potential impact of specific AS events on the prognosis of LGG patients. Finally, we investigated how SF gene-regulated AS events are involved in the occurrence and development of LGG. The purpose of this study was to explore the role of various splicing modes in LGG and to examine how SF genes regulate AS events and thus have an important role in the treatment and prognosis of LGG. The results of our study will provide new ideas for the treatment and detection of LGG

## RESULTS

**Figure 1:**
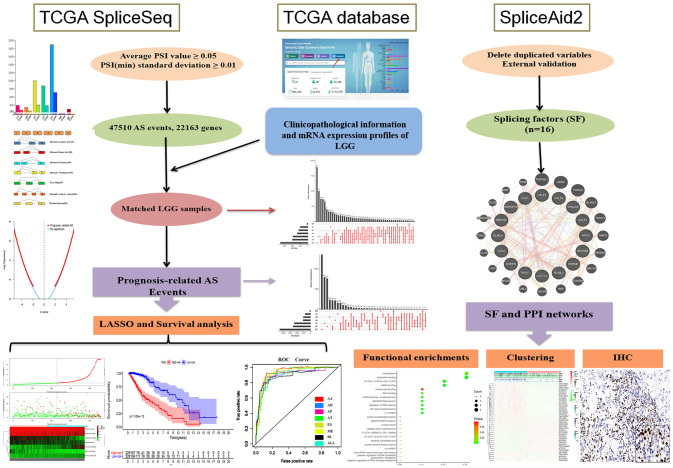
**Flowchart of the systematic profiling of the alternative splicing in glioblastoma multiform in this study.** TCGA, the Cancer Genome Atlas; AS, alternative splicing; LGG, low grade glioma; SF, splicing factor.

Our research was divided into three phases in sequence, and Figure 1 shows all processes of the system program. First and foremost, 47,510 prognosis-related AS events and 22,163 corresponding genes in LGG patients were identified after combining TCGA splice-sequence data and clinicopathological information with stringent filters. Then, survival analyses were performed after LASSO regression analyses of AA, AD, AP, AT, ES, ME, RI, and all types of AS events. Finally, we identified the SF genes and performed a detailed analysis of the regulatory role of these genes in AS events as well as the role they played in the occurrence and development of LGG.

### Integrated AS LGG group activities

**Figure 2:**
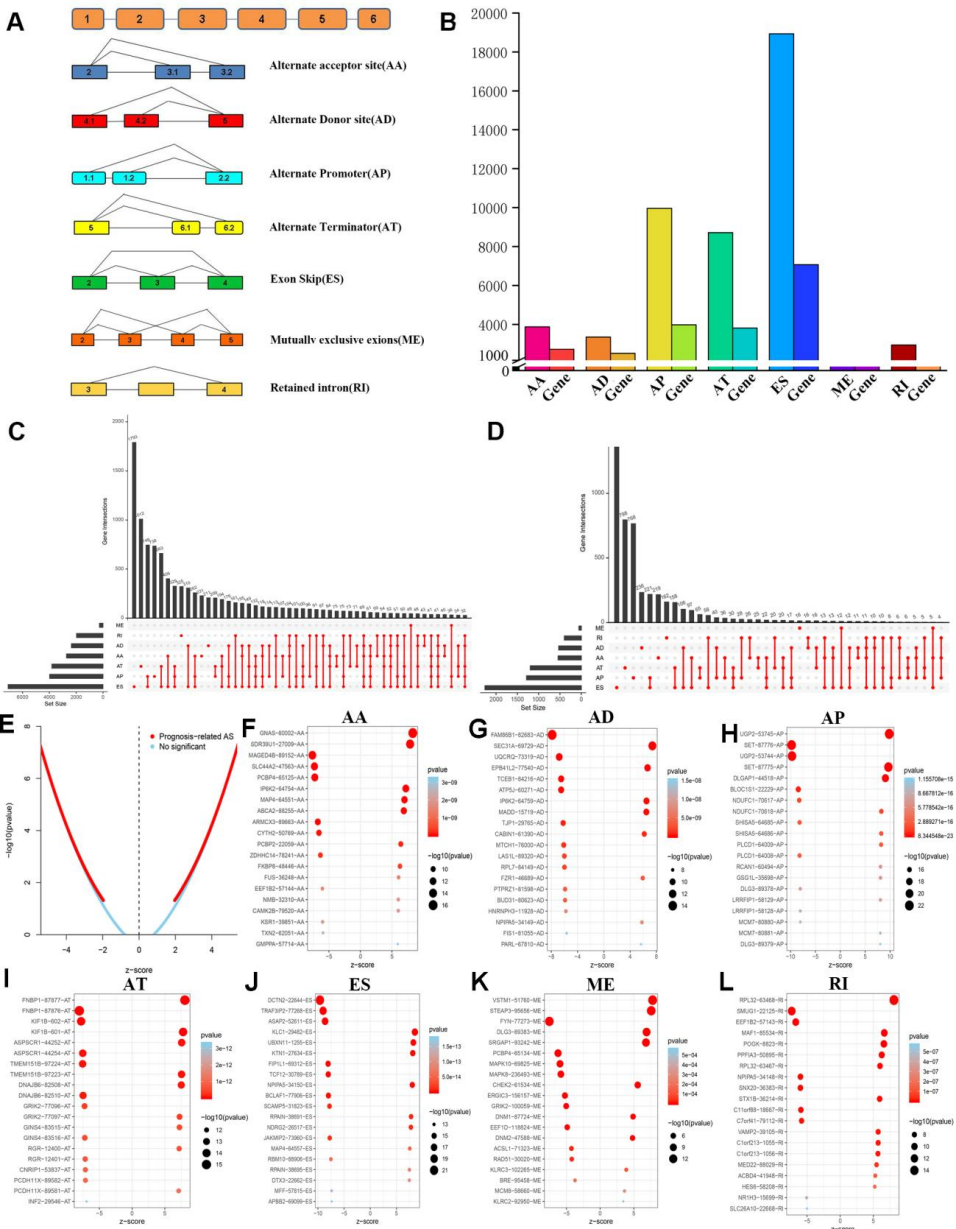
**Overview and identification of prognosis-related AS events of LGG.** (**A**) Schematic diagram of 7 different types of AS events was displayed. (**B**) 45,710 AS events in22,163 genes were obtained from 516 LGG patients after quality control. (**C**) Upset intersection diagram suggested that most genes might contain several kinds of AS events. (**D**) The Upset intersection diagram includes 7 different types of prognosis-related AS events in LGG. (**E**) Screening and identification of prognosis-related AS events and no significant AS events. (**F**–**L**) The top 20 significant prognosis-related AS events in 7 different types of AS events were illustrated in bubble charts.

A total of 47,510 AS events with 22,163 genes were determined in 516 LGG patients, indicating that the average number of AS events involved in each gene was approximately equal to 4. Figure 2A shows all seven types of AS events. Among these types, ES had the largest number of events, and AP and AT also had many. In detail, we detected 18,931 ES events in 7,074 genes, 9,964 AP in 3,976 genes, 8,718 AT in 3,809 genes, 3,876 AA in 2,716 genes, 3,351 AD in 2,353 genes, 2,937 RI in 1,971 genes and 273 ME in 261 genes (Figure 2B).

In LGG, ES is the most frequent AS event and ME is the rarest. Of note, a gene may have several different splicing patterns. We described the detailed information of genes of a specific AS type in the Upset diagram (Figure 2C). Compared with a traditional Venn diagram, it can more effectively prove the quantitative results of multiple interaction sets.

### LGG survival association with AS events

To investigate the prognostic value of AS events in LGG, we used Cox univariate survival analysis to assess the overall influence of every outcome of AS events.

Importantly, one gene may be involved in multiple survival-related AS events in LGG. Therefore, the UpSet graph illustrates the number of all types of AS events in LGG and a subset of overlapping AS events **(**Figure 2D). Furthermore, most genes are associated with at least two AS events, and some genes have a relationship with four AS events.

Therefore, we screened 9,616 AS events that were significantly associated with OS in LGG patients (*P*<0.05). We drew a volcano map based on the obtained data, which clearly shows that AS events relating to prognosis accounted for the majority (Figure 2E). In Figure 2F–2L, the 20 most important AS-related events with the highest survival rates among these seven types are shown, and most of these AS events are poor prognostic factors.

### LASSO regression analyses

**Figure 3:**
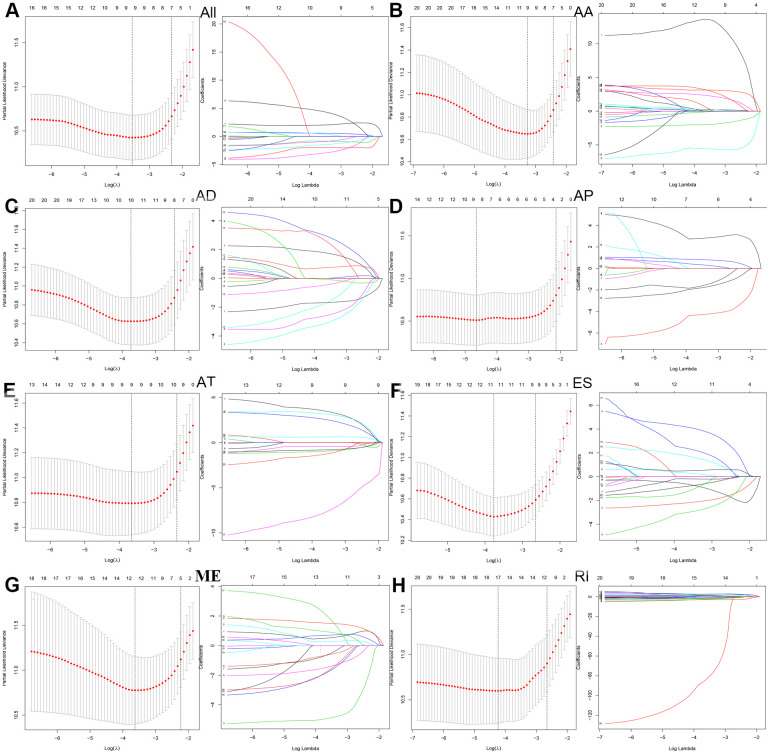
LASSO regression analyses were then performed in prognosis-related AS events in (**A**) all types of AS events, (**B**) AA, (**C**) AD, (**D**) AP, (**E**) AT, (**F**) ES, (**G**) ME and (**H**) RI.

Next, we performed a LASSO regression analysis of AA, AD, AP, AT, ES, ME, RI, and all types of AS events related to prognosis (Figure 3). The left mode of each group is the LASSO coefficient curve, and the right mode of each group is the choice of the adjustment parameter (λ) in the LASSO model. The y-axis represents partial likelihood deviations. The lower x-axis indicates the logarithm (λ), and the upper x-axis represents the average number of predictors. The red dot represents the average deviation value for each model with a given lambda, where the location announced the best data. As a result, the prognostic-relevant AS events in the eight groups were selected, including 12 AA events, 10 AD events, 10 AP events, nine AT events, 12 ES events, 13 ME events, 18 RI events, and 10 all-AS events.

### Prognostic factors of LGG queue

**Figure 4:**
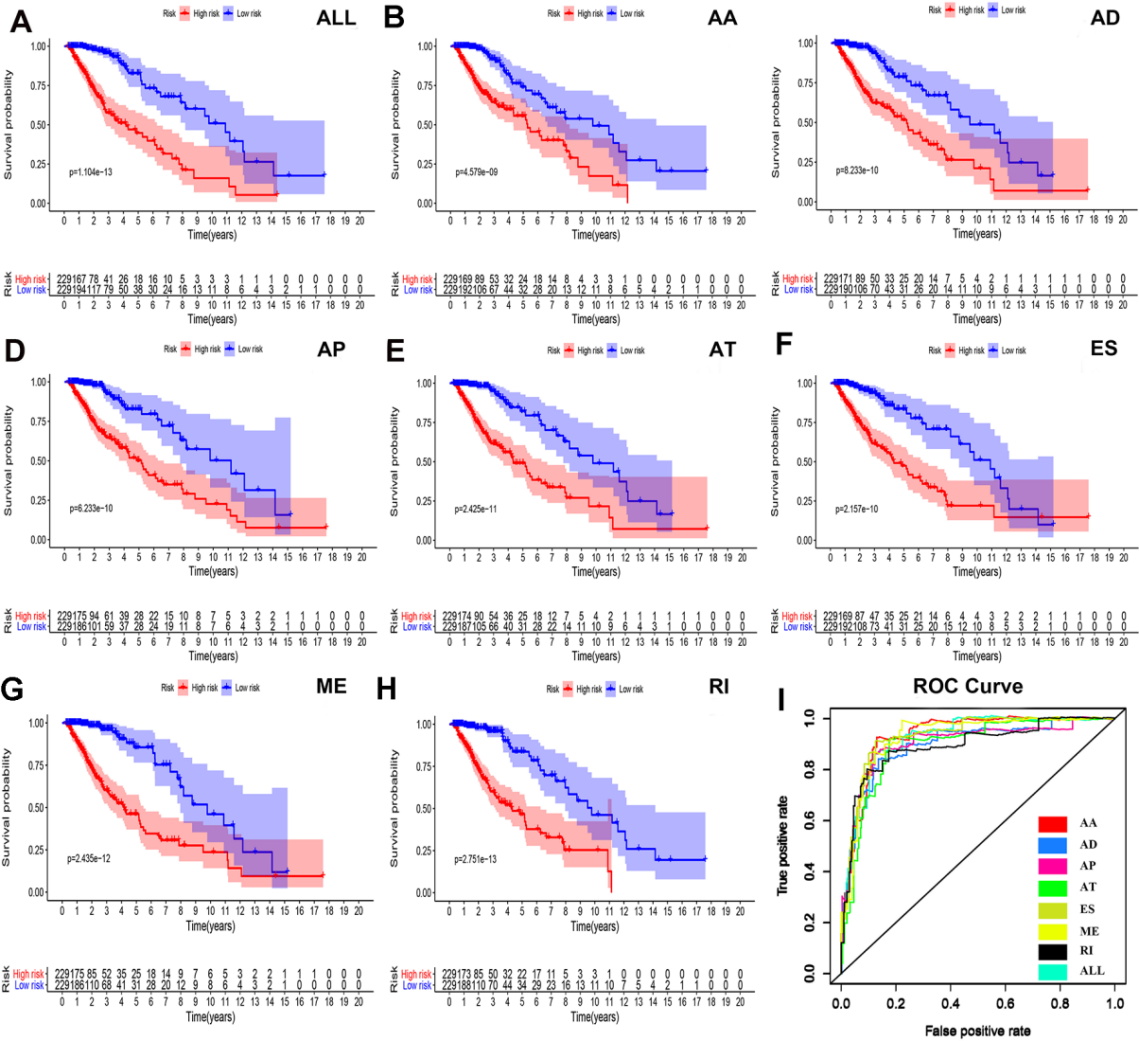
**Survival analyses and ROC constructions of prognosis-related AS events.** The risk score of each LGG patient was calculated using multivariate Cox regression analyses after LASSO regression analyses. The cut-off of low- and high-risk groups was decided by the risk score, and Kaplan-Meier survival analyses were presented in each group of (**A**) all types of AS events, (**B**) AA, (**C**) AD, (**D**) AP, (**E**) AT, (**F**) ES, (**G**) ME and (**H**) RI. (**I**) The final prognostic index ROC has an AUC of 0.924(0.891-0.934).

To determine the independent prognostic factors associated with LGG patients in AS events, we screened the most important AS events. A multiple Cox regression model with independent prognostic factors was performed for all other AS events. To confirm the final prognostic factors, we analyzed and screened all different types of candidate events and independent prognostic AS events. In our data analysis, we found that in all splicing modes, among the prognostic models composed of different types of AS events, all models had a strong ability to predict the prognosis of LGG patients (Figure 4A–4H).

We were not satisfied with this result, so we established a single prognostic model showing the capabilities of each of the seven strongest prognostic models (Figure 4B–4H). In addition, we aggregated independent prognostic AS events for seven different candidates to establish a final prognostic indicator. It is worth noting that the final prognostic indicators show better prognostic indicators than some single types of splicing mode (Figure 4H). The final prognostic index ROC had an AUC of 0.924 (0.891–0.934). The highest AUC was AA (0.934), followed by the ES (0.931) and ME (0.926) models (Figure 4I).

### Survival risk assessment and Cox regression analyses

**Figure 5:**
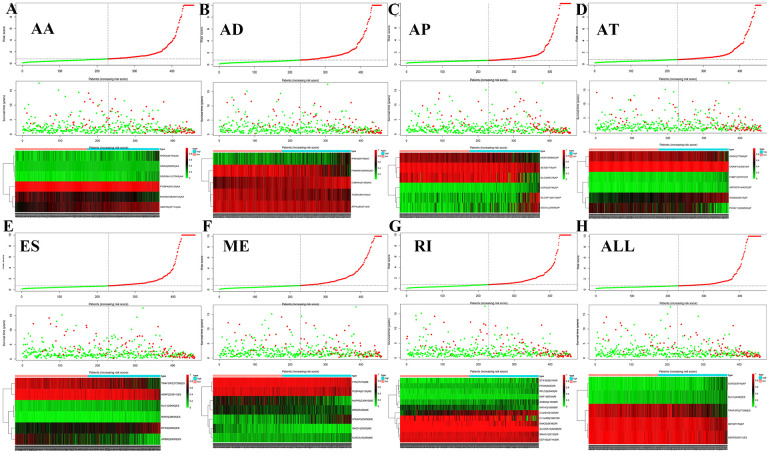
**Survival risk assessment of prognosis-related AS events.** The distribution of survival time, status and risk score in low- and high-risk groups were shown in all types of AS events, (**A**) AA, (**B**) AD, (**C**) AP, (**D**) AT, (**E**) ES, (**F**) ME, (**G**) RI and (**H**) all types of AS events.

Figure 5 shows the distributions of survival time, status, and risk scores among the low-risk and high-risk groups. The image is divided into upper, middle, and lower sections. The top portion indicates the distribution of risk scores, the middle part shows the survival time and status of LGG patients (green indicates alive, red indicates dead), and the bottom part is the heatmap associated with it. The black vertical dashed line in the middle indicates the best dividing line between the low-risk group and the high-risk group. AS events (Figure 5A) including ES of *KLC1*, ES of *TRAF3IP2*, and AP of *UGP2*, were associated with good prognosis. AS events including AP of *SET* and ES of *ASAP2* were associated with poor prognosis.

**Figure 6:**
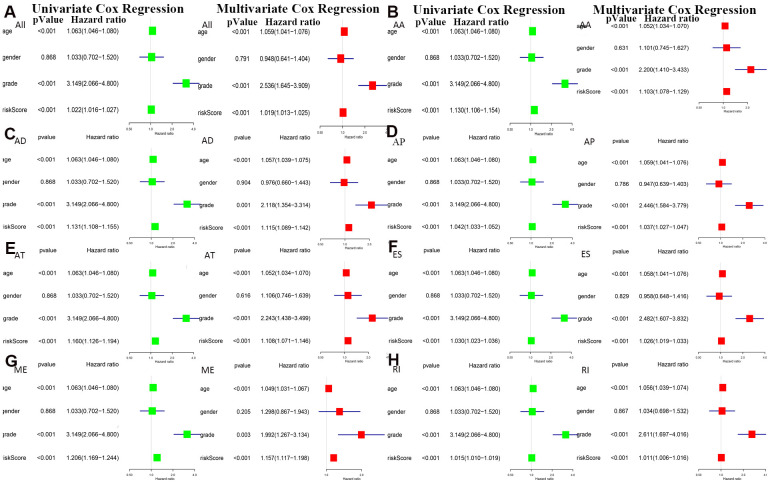
****Univariate and multivariate Cox regression analyses of prognosis-related AS events in 8 groups were performed in forest plots of (**A**) all types of AS events, (**B**) AA, (**C**) AD, (**D**) AP, (**E**) AT, (**F**) ES, (**G**) ME and (**H**) RI (*P* < 0.05).

The bottom section shows a heatmap of the PSI values of the corresponding final AS predictors. As shown in Figure 6, we executed univariate and multivariate Cox regression analyses of the eight groups of prognosis-related AS events in the forest plot. We analyzed the data using univariate Cox regression, showing that age (reference low), grade (reference low), and risk score (reference low) of the eight groups were used as independent prognostic indicators of LGG (*P*<0.05). Multivariate Cox regression analyses also demonstrated that age (reference low), grade (reference low), and risk score (reference low) of the eight groups were independent prognostic indicators of LGG (*P*<0.05).

### Correlation analyses between AS and SF genes

**Figure 7:**
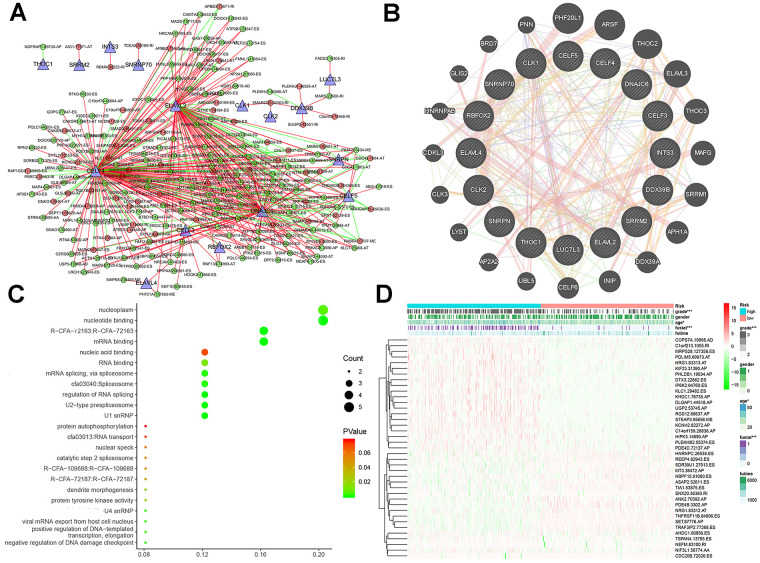
**Interaction networks and functional annotations of SFs.** (**A**) A total of 16 SF genes were identified to be significantly related with the regulation of AS events in LGG patients. (**B**) The PPI network of 16 SFs and neighbor protein nodes. (**C**) The results of GO analyses of SFs. (**D**) Overall heat map of AS events related to LGG survival.

Finally, we compared the identified SF genes with the genes obtained from TCGA and extracted the expression data of SF genes from the latter. We screened a total of 16 SF-related genes and constructed a regulatory network of AS and SF genes (IcorI> 0.8, *P*<0.001) using Cytoscape, and integrated the analyses with the risk factors (Figure 7A). In related networks, there were 16 survival-related SF genes (blue triangles) and 207 survival-related AS events, including 146 good (low-risk) AS events (green circles) and 61 bad (high-risk) AS events (red circles). We used line colors to indicate mutual relationships: red indicates positive feedback, green indicates negative feedback.

As illustrated in Figure 7B, gene–protein interaction analyses of 16 SFs and related genes were performed. Different line colors represent different types of gene–gene interaction networks: 49.56% were physical interactions, 22.29% were co-expression, 13.41% were predicted, 8.57% were co-localization, 6.06% shared protein domains, and 0.11% were genetic interactions. In Figure 7C, we executed a functional and pathway enrichment analysis of the 16 SF genes, and the results were displayed in bubble charts. The results of GO analyses showed that changes in BPs of SFs were significantly enriched in RNA processing and mRNA processing, changes in CCs of SFs were mostly enriched in RNA binding and mRNA binding, and changes in MFs of SFs were mostly enriched in spliceosomal snRNP complexes and U2-type prespliceosomes. Changes in Reactome pathway enrichment analyses existed in mRNA 3'-end processing and post-elongation processing of intron-containing pre-mRNA. Changes in KEGG pathway enrichment analyses existed in spliceosome and RNA transport.

### Subsistence analysis

**Figure 8:**
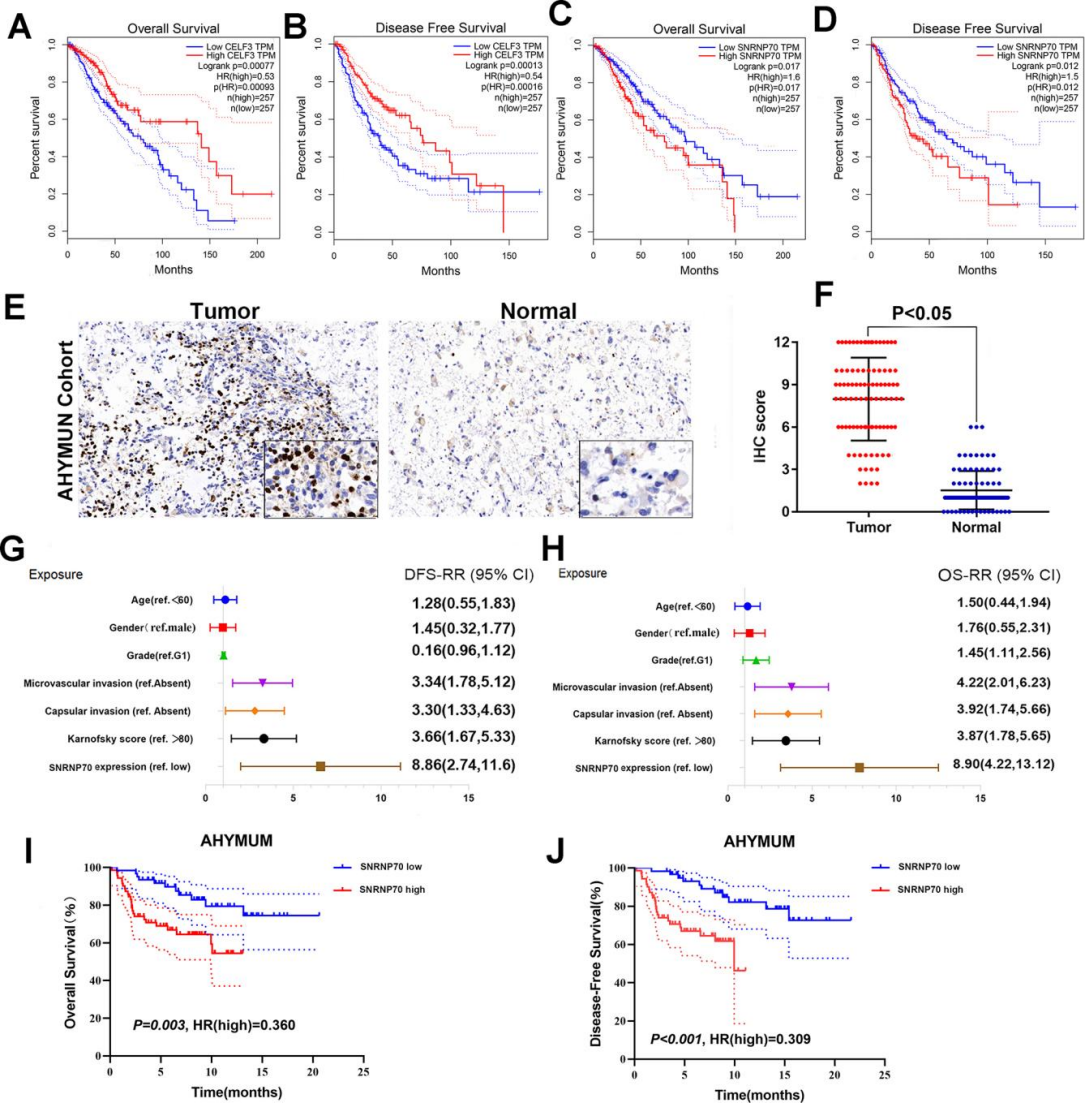
**Further verification of SF gene and LGG prognosis.** (**A**–**D**) OS and DFS of two example genes in LGG. CELF3 (**A**, **B**), SNRNP70 (**C**, **D**); (**E**) IHC on collected LGG tissue; (**F**) The scatter plot of the IHC scores(*P* < 0.05); (**G**, **H**) Forest plots were used to visualize the univariate Cox regression analysis of DFS and OS in the AHYMUM cohorts. (**I**, **J**) Survival curves showed that LGG patients with elevated SNRNP70 expression levels in the AHYMUN cohort showed poorer OS (*P* = 0.003) and poorer DFS (*P* < 0.001).

A heatmap (Figure 7D) of AS events and clinical correlations was generated showing the correlation between various clinical indicators and AS events and SF genes. Considering that all eight groups of AS events were related to the prognosis of LGG patients, the SF genes were related to the prognosis of LGG patients and were either positively correlated, negatively correlated, or balanced. Based on the constructed AS–SF network, we further studied how the SF genes regulate AS events, thereby affecting the treatment and prognosis of LGG. We analyzed the datasets in the Oncomine database to verify the effects of these genes on the OS and DFS of LGG. We examined the relationship between all 16 genes and LGG survival prognosis. In this network, we can clearly see what role SF genes play in AS events and how they affect the prognosis of LGG by regulating AS events. For example, *CELF3* was positively correlated with good AS events and negatively correlated with adverse AS events; after searching, we found that it was indeed negatively correlated with the poor prognosis of LGG (Figure 8A, 8B). As another example, we found that *SNRNP70* was positively correlated with adverse AS events, suggesting that this gene is positively correlated with LGG poor prognosis. We validated it with OS/DFS and demonstrated that *SNRNP70* is indeed associated with a poor prognosis of LGG (Figure 8C, 8D).

As another example, we found that *SNRNP70* was positively correlated with adverse AS events and negatively correlated with good AS events, suggesting that this gene is positively correlated with LGG poor prognosis. We validated it with OS and demonstrated that *SNRNP70* is indeed associated with a poor prognosis of LGG.

### Clinicopathological features related to SF expression statuses in the cohort

To verify our hypothesis, we performed immunohistochemistry (IHC) to reveal the staining distribution in tumor and normal tissues (Figure 8E, 8F). The scatter plot of the IHC scores revealed that *SNRNP70* expression was significantly elevated in LGG tissues in the AHYMUN cohort (*P*<0.001). We then selected all LGG patients from 2003 to the present. As shown in Table 1, in the AHYMUN cohort, SF expression increased with age (*P*=0.022), increased grade (*P*=0.009), history of epilepsy (*P*=0.045), and a lower Karnofsky score (which also indicates that the patient is less tolerant to the next treatment) (*P=*0.002). The chi-squared test showed that the baseline data were balanced in the distribution of the categorical data, including sex, incidence of microvascular invasion, and presence of capsule infiltration (*P*>0.05).

**Table 1:** Clinicopathological characteristics in relation to SNRNP70 expression level in AHYMUM cohort.

**Characteristics**	**AHYMUM cohort**	**SNRNP70 expression**		**χ^2^**	***P***
	**(N=100)**	**Low IHC score**	**High IHC score**		
		**(N=50)**	**(N=50)**		
N (%)					
**Age**				5.263	**0.022**
<60 years	81(0.81)	36(0.44)	45(0.56)		
≥60 years	19(0.19)	14(0.74)	5(0.26)		
**Gender**				2.21	0.137
Male	87(0.87)	41(0.47)	46(0.56)		
Female	13(0.13)	9(0.69)	4(0.31)		
**Grade**				6.775	**0.009**
G1	82(0.82)	46(0.56)	36(0.44)		
G2	18(0.18)	4(0.22)	14(0.78)		
**Seizure history**				4.336	0.066
yes	82(0.82)	45(0.55)	37(0.45)		
no	18(0.18)	5(0.28)	13(0.72)		
**Microvascular invasion**				10.746	**0.001**
Absent	76(0.76)	31(0.41)	45(0.59)		
Present	24(0.24)	19(0.79)	5(0.21)		
**Capsular invasion**				0.271	0.063
Absent	82(0.82)	40(0.49)	42(0.51)		
Present	18(0.18)	10(0.56)	8(0.44)		
**Karnofsky score**				9.458	**0.002**
≥80	61(0.61)	38(0.62)	23(0.38)		
<80	39(0.39)	12(0.31)	27(0.69)		

### Cox regression analysis

As shown in the forest plot, univariate Cox regression analysis showed that there was no significant correlation between sex and epilepsy history in patients from AHYMUN (Figure 8G) and DFS. In the multivariate model, the Karnofsky score (ref. <80) was significantly correlated with DFS in LGG patients (HR=1.914; *P=*0.045). More importantly, a subgroup analysis of *SNRNP70* expression showed that in this cohort, amplification of *SNRNP70* was significantly correlated with DFS (HR=3.048; *P=*0.001). In addition, according to the multivariate model used for Cox regression analysis, in the AHNTU cohort, the presence of capsular infiltration was significantly associated with poor DFS (HR=2.66; *P=*0.006), and microvascular invasion was also significantly associated with poor DFS (HR=2.563; *P=*0.007) (Table 2).

**Table 2:** Multivariate Cox regression analysis of DFS and OS in AHYMUM cohorts (DFS: disease-free survival; OS: overall survival).

**Covariates**	**DFS**				**OS**		
	**HR**	**95% CI**	***P* value**		**HR**	**95% CI**	***P* value**
Grade (ref. G1)	0.822	0.412-1.707	0.609		0.915	0.412-2.443	0.832
Microvascular invasion (ref. Absent)	2.563	1.286-5.110	**0.007**		2.826	1.186-5.712	**0.024**
Capsular invasion (ref. Absent)	2.660	1.331-5.317	**0.006**		2.691	1.231-6.617	**0.044**
Karnofsky score (ref. >80)	1.914	1.014-3.611	**0.045**		2.127	1.176-4.927	**0.031**
SNRNP70 expression (ref. low)	3.084	1.632-5.828	**0.001**		6.246	1.596-16.424	**<0.001**

Several parameters such as sex and epilepsy history in the cohorts in Figure 8H were not significantly related to OS. As shown in Table 2, multivariate Cox analysis showed that Karnofsky scores (ref <80) were significantly correlated with OS in the AHYMUN cohort (HR=2.127; *P=*0.031). SF gene amplification was significantly associated with OS in AHYMUN patients (HR=6.246; *P*<0.001). Other factors, including capsular infiltration and microvascular invasion, were related to OS (*P*<0.05).

Survival curves showed that LGG patients with elevated *SNRNP70* expression levels in the AHYMUN cohort showed poorer OS (*P=*0.003) and poorer DFS (*P*<0.001) (Figure 8I, 8J).

## DISCUSSION

In this study, we first correlated survival-related AS events in LGG with SF genes. We analyzed the correlation between AS and SF in LGG. We used the SF genes to predict the development and prognosis of LGG patients and confirmed our hypothesis through OS and DFS. Our splicing network constructed by AS events and SF genes further lays the foundation for the regulatory mechanism of LGG occurrence and development. Survival-related AS in this study provides new ideas for new targeted therapies for LGG.

Gliomas are the most common brain tumors and are characterized by rapid growth and early relapse. Patients usually have a poor prognosis [15]. Diffuse gliomas account for most intracranial malignancies, comprising more than 60% of cases [6]. Low-grade glioma (LGG) is a slow-growing solid invasive primary brain tumor (WHO I–II) [16, 17]. At the beginning, its clinical manifestations are not obvious and many patients consult a doctor for seizures [18–20]. Despite the development of various treatments, LGG cannot be cured. As tumors develop [21, 22] and gradually invade the central nervous system, LGG inevitably progresses to HGG, leading to poor prognosis [23–25]. To date, biomarkers that can effectively reflect the prognosis of LGG are lacking.

AS is common in mRNA processing and has an important role. It can cause a variety of complex changes in proteins, relying on a limited genome, and resulting in a large number of proteins [26]. Much research has given us a better understanding of the role and changes of AS in cancer. In tumors, abnormal AS can occur in a variety of ways, making it difficult to elucidate the role of each AS event in cancer [27–30].

Studies have found that some mRNAs associated with AS events are related to the development of cancer [31]. Some researchers have started to correlate AS with the development of cancer subtypes to explore its impact on cancer prognosis [7], such as that of prostate [32], ovarian [33], and colorectal cancer [34]. Several studies have applied AS to the clinical practice of assessing cancer prognosis, providing new ideas for assessing tumor prognosis at the molecular level. Thus far, LGG has not been examined with respect to AS. In our study, we not only screened AS events related to LGG survival, but also established a model to predict the survival of LGG patients after surgery. In addition, we also screened SF-enriched genes and analyzed AS co-expression in the protein–SF network to further investigate AS molecules in LGG and the molecular mechanisms underlying SF genes.

We analyzed data for all the splicing patterns studied, and from these data we found that all subgroups of AS patterns have clinical survival advantages. The observations from this study indicate that one gene can produce multiple mRNAs through AS, resulting in numerous transcription events to produce many proteins. Our study found that these have more or less of an impact on the prognosis of LGG. For example, *CLK2* was only positively related to the interaction of bad AS events, so it had a negative impact on the prognosis of LGG. Therefore, this is not conducive to the recovery of LGG, and the likelihood is that LGG recurrence will increase in patients with high expression. *ELAVL2* is negatively correlated with adverse AS events and positively correlated with good AS events; therefore, it has a positive impact on the prognosis of LGG. Not only is the prognosis good, but the likelihood of relapse is reduced. The relationship between SF genes such as *DNAJC6* and *CELF5* and AS events is complex, and the impact of these core genes on the occurrence and development of LGG requires further study. However, these SF genes may be potential biomarkers of LGG and may play an important role in determining patient treatment options and observing prognosis. We also selected 100 LGG patients from the tissue bank of our hospital to verify our hypothesis.

This study has some limitation. First, although we analyzed the correlation between the SF genes and AS, their mechanism of action and regulatory methods have not been fully determined, and the role of other regulatory factors in LGG is unclear. Therefore, more research is needed to clarify the prognostic value of comprehensive splicing regulatory networks and regulatory factors. Second, we only selected cases from our center for retrospective research, and we will expand the sample size and range in the next study.

In conclusion, we not only established data on overall AS-related events in LGG, but also found that the prognostic markers identified in AS events showed satisfactory predictive effects for the survival of LGG patients. Comprehensive genome-wide profiling of the AS landscape in our LGG cohort revealed novel AS patterns associated with carcinogenesis and aggressive progression, which may shed light on AS-related clinical implications in LGG. This work further elucidated SF gene-regulated AS events, laying the foundation for the future molecular treatment of LGG and providing novel perspectives on LGG treatment and prognostication. We have not studied whether there are some prognostic AS patterns or splicing factors that are common to all tumors, which will be our new point of inquiry. In addition, merely expression level and prognostic value of SNRNP70 were identified in this study, thus further functional works, as well as in-depth mechanisms were needed to verify the absoluteness of these findings.

## MATERIALS AND METHODS

### Collection and standardization of data

We downloaded mRNA replacement splicing data for 516 LGG patients from the TCGA SpliceSeq dataset (bioinformatics.mdanderson.org) [35]. We then performed a normalized test on these data using percent spliced in index (PSI) values. The average PSI value of each AS event was greater than 0.05, and the minimum PSI standard deviation was greater than 0.01. We then performed an interactive quantitative analysis of all seven AS event types in the form of an UpSet chart [36]. At the same time, we obtained the RNA sequencing data and clinicopathological information of 516 LGG patients from TCGA database [37], and merged it with mRNA alternative splicing data. In addition, to identify the potential correlation between SF genes and prognosis-related AS events, we obtained a list of SFs from the SpliceAid2 database [38].

We downloaded RNA-seq data from the LGG cohort of TCGA data portal (https://portal.gdc.cancer.gov/projects). The PSI value quantifies AS events and calculates the ratio of the seven types. In our research, we not only analyzed the seven AS events individually, but also analyzed the overall AS events. Because the PSI value represents the percentage of standardized read counts that include the transcript element in all standardized reads (including reads and exclude reads) of the event, a change in the average PSI value indicates that the splicing pattern between groups has changed.

In this study, from January 2003 to December 2019, 100 LGG patients were recruited from the Affiliated Hospital of Youjiang National Medical University (AHYMUN) (Baise, China). The inclusion criteria included: (1) pathology (surgery or biopsy) and typical dynamic imaging examination; (2) no tumor treatment or tumor resection has been performed before; and (3) no other brain tumors. The clinical and pathological parameters of our cohort included age at surgery, sex, grade, seizure history, Karnofsky score, degree of microvascular invasion, and capsule invasion. Tissue samples including LGG and adjacent normal tissues were collected during surgery and these samples were obtained from the AHYMUN tissue bank. All study design and testing procedures were performed in accordance with Helsinki Declaration II. Ethical approval was obtained from the ethics committee and all patients provided consent to participate in this study.

### Identification of prognosis-related AS events

We screened AS events related to prognosis based on the PSI values and OS of LGG patients and analyzed these date by univariate Cox regression analysis. We performed a quantitative intersection analysis on the seven different prognosis-related AS events and displayed them in the form of an UpSet graph. Then, we used bubble charts to show the top 20 important events in the seven different AS events. We used the glmnet software in R (version 3.6.2) to perform a LASSO regression analysis of all AS events to screen the most important AS events related to prognosis, and also to avoid overfitting. After that, we performed multivariate Cox regression analysis on the obtained data and calculated the risk score of each LGG patient based on the corresponding highly important prognosis-related AS PSI value. Finally, we divided the 516 confirmed LGG patients into low-risk and high-risk groups based on risk scores.

### Survival analysis

This study included 516 patients with complete clinical parameters, all of whom were LGG patients. We performed a Cox univariate analysis to assess all other splicing events that may affect OS in LGG patients and analyzed their clinical significance. We also performed a multivariate Cox analysis of all types of AS events to determine their prognostic factors. We combined all independent prognostic AS events of the seven different types to establish the final indicators of prognosis. In addition, we plotted Kaplan-Meier curves as a prognostic indicator for patients with LGG OS over 5 years. We used the receiver operating characteristic (ROC) software package (version 1.0.3) of the R software to compare the survival efficiency of each prediction model [39].

### Comprehensive bioinformatics and statistical analysis

We distinguished the intersections between AS events that occurred in LGG and related survival, and visualized the results using Upset charts. In Cytoscape software (version 3.7.2), we entered the gene names related to the survival prognosis of LGG patients [40]. By analyzing the constructed gene network, we identified the most critical central genes, and thus established a SF network related to the prognosis of LGG. The relationship between crucial SF gene expression and AS events was established. Finally, we used Cytoscape to construct the relevant pictures. All statistical analyses were performed using BiocManager (version 1.30.1), and all *P*-values of <0.05 were considered statistically significant (*P*-values are two-sided). To investigate the difference in AS between LGG tissue and normal brain tissue, we calculated the percentage of all AS types. To accurately evaluate the value of AS in predicting the occurrence, development, and prognosis of LGG, we use ROC curves to describe it. In addition, we analyzed the effect of AS events on OS and disease-free survival (DFS) of LGG patients by the Cox model to screen for possible prognostic biomarkers. To further explore the independent clinical factors related to LGG, we performed univariate and multivariate Cox regression analyses on age (reference low), gender (reference male), grade (reference g1), microvascular invasion (reference absent), capsular invasion (reference absent), Karnofsky score (reference > 80) and SF gene expression (reference low) of the eight groups in the AS events analysis.

### Prognosis of AS and feature-rich analysis of SF

We identified the functional enrichment of SFs in biological attributes by gene ontology (GO) enrichment analysis, including biological processes (BPs), cellular components (CCs), and molecular functions (MFs) in the form of bubble diagrams. SF analysis was also performed for the pathway enrichment analysis of the reaction group, and other related pathways that were important in the Reactome analysis were also introduced in detail. GO enrichment analysis of SFs was further illustrated and visualized by ClueGO (version 2.5.4) and CluePedia (version 1.5.4), which are Cytoscape plug-ins that are used to visualize non-redundant biological terms of gene modules in functional grouping networks.

### Correlation analysis of prognosis-related AS and SFs

The correlation among SFs and the eight groups of AS events was displayed in a heatmap. Then, hierarchical clustering of the identified SFs was added to the heatmap based on the mRNA expression of SFs in 516 LGG patients. TCGA Splicing Variants Database was used to identify splicing locations on the exons and junctions of some genes.

### Statistical analysis

R (Version 3.6.2) and RStudio (Version 1.2.1335) were utilized to complete an Upset plot, univariate and multivariate Cox regression analyses, LASSO regression analyses, Kaplan-Meier plots, ROC curves, risk plots, PPI network, and functional annotations. In all tests, both sides and *P*-values less than 0.05 were considered significant.

To determine the relationship between different SF gene expression levels and clinicopathological features, a χ2 test was performed to compare the distribution of classification data between groups. A scatter plot was used to represent the differential expression of SNRNP70 in normal and LGG tissues. The primary endpoint was the OS of patients who survived a specific period of time, which was determined based on the length of time from the date of surgery to the date of death or the date of the last follow-up. DFS as a secondary endpoint refers to the length of time from the start of curative treatment for which no disease can be found to the date of progression, the date of starting second-line treatment, death, or whichever occurred first. The follow-up time was estimated using the Kaplan-Meier method (95% confidence interval) (95% CI) and log-rank test (with independent curve). Hazard ratios were derived from Cox proportional hazard regression models based on high-to-low comparisons to identify independent predictors. Univariate and multiple Cox regression models were independently analyzed to assess the effects of confounding variables including age at surgery, sex, grade, history of epilepsy, Karnofsky score, microvascular invasion, capsular invasion, and gene expression. Statistical analysis was performed using SPSS software (version 23.0, SPSS Inc., Chicago, Illinois, USA). All hypothesis tests are two-sided tests, and all tests have *P*-values of less than 0.05.

### Immunohistochemistry

Immunohistochemical streptavidin-peroxidase method was used to detect the expression of SFs in LGG and adjacent tissues (we stained every SF gene we screened). The tissue was cut into sections with a thickness of about 5 μM, then dewaxed, hydrated and immersed in hydrogen peroxide to inactivate endogenous peroxidase. Catalase, microwave repair with 0.01 mol / L sodium citrate buffer, I anti-blocking, II anti-incubation, dropwise addition of DAB colored solution, rinse with tap water, and counterstain with hematoxylin, dehydrated, transparent 2. The slides were sealed and then sealed under a microscope. Pathological sections were judged by two senior pathologists. Five high power field of view were randomly selected on each slide and brownish-yellow particles were considered as positive expression. Samples were scored based on cell staining (0 to 3 points): 0, cytosolic yellow particles; 0, cytosolic yellow particles. 1. Light brownish yellow particles are higher than background and negative control; 2 are obviously brownish yellow particles; 3. Stained by a large number of dark brown particles. Samples were also scored based on the percentage of positive cells (0 to 4 points): 0% was rated as 0 points, <10% was rated as 1 point, 11% to 50% was rated as 2 points, and 51% to 80%. Rated 3 points and> 80% rated 4 points. Calculate the immune response score (IRS) by multiplying the two scores [41].
